# Budd-Chiari Syndrome: Shunt or Transplant?

**DOI:** 10.1155/1994/24252

**Published:** 1994

**Authors:** Paul McMaster

**Affiliations:** Liver & Hepatobiliary Unit Queen Elizabeth Hospital Edgbaston Birmingham B15 2TH UK


3. Shinagawa, T., Ohto, M., Kimura, K., *et al.* (1984) Diagnosis and clinical features of small hepatocellular carcinoma with emphasis on the utility of real-time ultrasonography: a study in 51 patients. *Gastroenterology*, **86**, 495–502
4. Colombo, M., DeFranchis, R., Del Ninno, E., *et al.* (1991) Hepatocellular carcinoma in Italian patients with cirrhosis. *N.Engl.J.Med.*, **325**, 675–680
5. Okuda, K., Fujimoto, I., Hanai, A. and Urano, Y. (1987) Changing incidence of hepatocellular carcinoma in Japan. *Cancer Res.*, **47**, 4967–4972
6. Colombo, M., Kuo, G., Choo, Q.L. *et al.* (1989) Prevalence of antibodies to hepatitis C virus in Italian patients with hepatocellular carcinoma. *Lancet*, **2**, 1006–1008
7. Okuda, K. (1992) Hepatocellular carcinoma: recent progress. *Hepatology*, **15**, 948-963
8. Edmondson, H.A. and Steiner, P.E. (1954) Primary carcinoma of the liver: a study of 100 cases among 48,900 necropsies. *Cancer*, **7**, 462–503
9. Ebara, M., Ohto, M., Shinagawa, T. *et al.* (1986) Natural history of minute hepatocellular carcinoma smaller than three centimeters complicating cirrhosis. *Gastroenterology*, **90**, 289–298
10. Calvet, X., Bruix, J., Gines, P. *et al.* (1990) Prognostic factors of hepatocellular carcinoma in the West. A multivariate analysis in 206 patients. *Hepatology*, **12**, 753–760
11. Sheu, J.C., Sung, J.L., Chen, D.S. *et al.* (1985) Growth rate of asymptomatic hepatocellular carcinoma and its clinical implications. *Gastroenterology*, **89**, 259–266
12. Nomura, F., Ohnishi, K. and Tanabe, Y. (1989) Clinical features and prognosis of hepatocellular carcinoma with reference to serum alpha-fetoprotein levels. Analysis of 606 patients. *Cancer*, **64**, 1700–1707

Kunio Okuda, MD, PhD.
First Department of Medicine
Chiba University School of Medicine
Chiba 260
Japan

### BUDD-CHIARI SYNDROME: SHUNT OR TRANSPLANT?

### ABSTRACT

*Shaked, A., Goldstein, R.M., Klintmalm, G.B., Drazan, K., Husberg, B. and Busuttil, R.W. (1992) Portosystemic shunt versus orthotopic liver transplantation for the Budd-Chiari Syndrome. Surgery, Gynecology & Obstetrics; 174: 453–459.*

**We have analyzed the indications and results of shunt operation versus orthotopic liver transplantation (OLT) in 22 patients with Budd-Chiari syndrome (BCS). The underlying cause of the syndrome was similar between the two groups and was related to myeloproliferative disorders or the use of birth control pills in 18 of 22 patients. The results of biopsies of the liver showed centrilobular congestion and necrosis in all candidates who underwent shunting and the presence of fibrosis and cirrhosis in the OLT candidates. The indications for shunts included symptoms related to portal hypertension only and well-preserved synthetic hepatic function. Ten patients were treated with 12 shunt procedures, including mesoatrial (eight patients) and side to side portacaval shunt (four patients). Significant complications**

**after shunt procedure included fulminant (one of ten patients) and progressive (one of ten patients) hepatic failure requiring urgent OLT; one death occurred because of pulmonary sepsis. Indications for OLT were signs of end stage liver expressed by severe portal hypertension and variceal bleeding (four of 14 patients), progressive encephalopathy (seven of 14 patients) and poor synthetic function (bilirubin >3 milligrams per deciliter in eight of 14 patients and albumin <3.0 grams per liter, or both, in ten of 14 patients). Fourteen patients were treated with 16 OLT, three patients had retransplantation for primary nonfunction graft (two of 14 patients) or chronic rejection (one of 14 patients). There were two early deaths in the group. With a follow-up period between two months to five years, 12 of 14 patients undergoing OLT are alive, fully functional and have normal liver function tests. Seven of ten patients who had shunts are alive, six are able to maintain normal activity and one has progressive end stage hepatic disease and is not a candidate for OLT. However, the hepatic function continues progressively to be abnormal.**

**Various options are available for the treatment of the syndrome. Portosystemic decompression is effective and should be considered at the early stage of the disease, prior to the development of significant hepatic failure. However, few of the patients will continue to have slow, but progressive hepatic failure and may require OLT. The only effective treatment for end stage hepatic disease secondary to the BCS is OLT.**

### PAPER DISCUSSION

KEY WORDS: Budd-Chiari Syndrome, liver transplant, portosystemic shunt

The Budd-Chiari Syndrome is fortunately uncommon as it affects relatively young patients who present with abdominal pain, ascites, hepatomegaly and deteriorating liver function. While mechanical outflow obstruction due to a vena-caval web, stricture or malignancy may produce the syndrome all too often a hypercoagulable state is responsible with myeloproliferative disorders such as polycythemia rubra vera in more than half of the cases with systemic lupus nocturnal haemoglobinuria and anti-thrombin III deficiency also implicated. The relative infrequency of such patients and small numbers attending an individual centre makes effective evaluation of treatment options difficult. The authors in their series outline the successes of liver transplantation in treating the chronic Budd-Chiari Syndrome and compare it with portal shunting. The European Liver Transplantation Registry currently records that patients grafted with Budd-Chiari Syndrome since 1988 will achieve a 3 year survival of over 75% (92 patients).

However, the shortage of available organs and the need for long term immunosuppression rightly means that alternative options should always be initially reviewed. Clearly late cases of Budd-Chiari Syndrome where there is advanced fibrosis and portal hypertension with cirrhosis will not be benefited by hepatic portal shunting and will probably require hepatic transplantation. However, in the early acute cases where fibrinolytic therapy has been ineffectve then shunting should be a clinical modality which is urgently reviewed. The development of massive hepatic congestion in the Budd-Chiari Syndrome but preserved hepatic function often can be resolved with adequate portal decompression by a portocaval shunt or alternatively a meso-atrial shunt when the cava is obliterated. The authors in their series report an experience of some 10 patients undergoing such shunting and report only one early death from pulmonary sepsis and two other cases of hepatic failure (one acutely and one as a slow progressive deterioration requiring transplantation.) The addition of Hydroxyurea and Aspirin prevented subsequent thrombosis although they are rightly concerned about the long term outcome and possible further hepatic deterioration. Nevertheless, very nearly three quarters of their patients were effectively salvaged in the short term by an urgent shunt avoiding the need for transplantation and long term immunosuppression.

It seems likely therefore that most patients with non-malignant Budd-Chiari Syndrome should be urgently considered for portal shunting. This has recetly been our own approach when no cirrhosis exists and we have tried whenever possible to avoid liver grafting and move at the earliest opportunity to porto-caval shunting if medical therapy (hepatic vein dilatation or fibrinolysis) is proving ineffective. This has clearly not been possible in the chronic Budd-Chiari patient with marked fibrosis, cirrhosis and hypertension. A similar number of acute patients were successfully shunted in our unit although usually using a meso-caval graft, success has been achieved in all but one who subsequently encountered a thrombosis to the graft.

The option of shunting however requires adequate hepatic reserve and the urgent assessment of the underlying liver is a critical step. Biopsies frequently need to be undertaken via the trans-jugular approach because of coagulation difficulties and the risk of bleeding following percutaneous biopsy.

If success is to be achieved then early shunting requires an early diagnosis and the essential clue perhaps to the early diagnosis is the disproportionate ascites in the face of relatively good liver function. The diagnosis is then confirmed by ultrasound of the hepatic veins followed by venography and trans-jugular biopsy. Urgent studies are needed to determine the underlying cause of the hypercoagulable state and bone marrow assessments looking for myeloproliferative disorders will usually be required.

The prompt diagnosis of the acute Budd-Chiari Syndrome offers a prospect of treatment perhaps initially by fibrinolysis or vein dilatation and then urgent consideration for meso-caval shunting. Liver grafting will usually be reserved for only those in whom shunting is followed by hepatic failure or in whom trans-jugular histology shows advanced fibrotic/cirrhotic changes. The use of the meso-caval shunt in no way comprises a subsequent liver transplant if that proves necessary when a relatively simple ligation of the shunt can be made, and also avoids the difficult enlarged caudate lobe seen in Budd-Chiari. Clearly however long term follow-up is now needed for these patients to assess if satisfactory long term hepatic function is achieved.

Paul McMaster
Consultant Surgeon
Liver & Hepatobiliary Unit
Queen Elizabeth Hospital
Edgbaston
Birmingham B15 2TH
England

